# Evaluation of non-endemic pemphigus foliaceus in a large series of patients: a single-center retrospective study from Turkey focuses on the relapses^☆^^☆☆^

**DOI:** 10.1016/j.abd.2020.12.009

**Published:** 2021-05-29

**Authors:** Sıla Kılıç Sayar, Rıfkiye Küçükoğlu

**Affiliations:** a Department of Dermatology and Venereology, Bahçeşehir University Faculty of Medicine, Istanbul, Turkey; b Department of Dermatology and Venereology, Istanbul Faculty of Medicine, Istanbul University, Istanbul, Turkey

**Keywords:** Pemphigus foliaceus, Relapse, Seasonal variation, Therapy, Ultraviolet

## Abstract

**Background:**

Pemphigus foliaceus is exceedingly rare around the world, except within the few regions where it occurs as an endemic variant. Various factors can trigger immune mechanisms that induce pemphigus foliaceus or worsen its course.

**Objective:**

To determine the demographic and clinical characteristics of the patients with pemphigus foliaceus in a large series from a non-endemic country, investigate the triggering factors, and seasonal patterns.

**Methods:**

The data of the patients diagnosed with pemphigus foliaceus in the study’s center between 1989–2018 were retrospectively analyzed.

**Results:**

Sixty-eight patients (mean age, 45.7 ± 14.5 years) were included in the study. The number of onsets reached its peak in spring-summer (p = 0.008). A total of 117 relapses occurred in 42 patients and were most common in spring-summer (not significant). Specific trigger factors were detected in 45 relapses. In the other 72 relapses, the peak was observed in spring-summer (p = 0.005). There were no significant differences in the demographic and clinical variables investigated between relapsed and non-relapsed patients.

**Study limitations:**

Retrospective design.

**Conclusions:**

Triggering factors could not be identified in more than half of the relapses in the study’s series. The subgroup of relapses (without identified causes), as well as the onsets of the disease, showed a significant seasonal variation with a peak in spring-summer; however, the seasonal variable did not justify the total group of relapses. Although the seasonal variation may be caused by a combination of factors, UV radiation should be considered a trigger factor for the peaks in spring-summer, particularly in Turkey.

## Introduction

Pemphigus is a group of autoimmune bullous skin diseases characterized by the autoantibodies directed against surface proteins of keratinocytes.^1^, ^2^ Pemphigus vulgaris (PV) and pemphigus foliaceus (PF) are the two main subtypes of pemphigus, and they exhibit clinical, histopathological, and immunological differences.^2^, ^3^ PF is very rare around the world, except within the few regions where it has an endemic form.^4^, ^5^, ^6^ Both variants of PF are characterized by superficial and fragile blisters that are seen on the histopathological evaluation as subcorneal acantholysis.^7^ This superficial pattern sometimes causes a wrong impression of the disease that it is a mild disease with an easily manageable course; however, various personal and exogenous factors were shown to trigger immune mechanisms that can cause multiple relapses in susceptible PF patients.^8^, ^9^ Ultraviolet (UV) radiation is one of the frequently investigated risk factors and it was reported to induce or exacerbate some autoimmune bullous diseases including PF, PV, linear IgA dermatosis, and bullous pemphigoid.^10^, ^11^, ^12^, ^13^, ^14^, ^15^, ^16^

Seasonal clustering patterns in many non-dermatological and dermatological diseases including PV have been previously reported.^17^, ^18^, ^19^, ^20^, ^21^ Since non-endemic PF is very rare, demographic, or other research on specific PF series from non-endemic countries are limited in the literature; however, the authors believe that both preventable trigger factors and seasonal patterns of relapses need attention in this disease. In this study, the authors aim to determine the demographic and clinical characteristics of PF in a large series of patients from a non-endemic country, as well as to investigate the possible personal and exogenous factors for relapses, and seasonal variations in the course of the disease.

## Methods

### Patients and setting

In this study, the authors retrospectively reviewed the files of patients who were consecutively diagnosed with PF in the authors’ tertiary referral center between 1989 and 2018. The patients, whose diagnosis had been confirmed by histopathological evaluation and at least one of the immunofluorescence techniques (direct immunofluorescence/indirect immunofluorescence), were enrolled in the study. Patient evaluation data included the following: age, gender, time delay prior to diagnosis, distribution of the lesions, smoking habit, comorbidities, professions, duration of follow-up period, the occurrence of relapse, and detected trigger factors of the relapses. The term “relapse” was adapted to the present study from a previous definition as the occurrence of new lesions that did not heal spontaneously within one week, or the extension of lesions in a clinically stable patient.^22^ Patients with a history of at least one relapse and those without were compared in terms of their demographic and clinical characteristics. Dates of the initial onset of symptoms and relapses were recorded by years, seasons, and months. The seasons are defined as winter (January, February, and March), spring (April, May, and June), summer (July, August, and September), and autumn (October, November, and December). Expected numbers in an equal distribution among seasonal groups (spring-summer/autumn-winter) were calculated and compared to observed numbers. Seasons were grouped in pairs in order to compare the times of the year that are with and without intense UV radiation according to the climate of Turkey.

### Statistical analysis

Statistical analysis was performed through IBM SPSS Statistics 22.0 (SPSS, Chicago, IL, USA). Continuous data were presented as means ± Standard Deviation (SD), median, and range. Intergroup differences were analyzed using binomial logistic regression for continuous and categorical variables. Odds Ratios (ORs) of the variables were measured by logistic regression analysis. The authors used a chi-square test to analyze whether there was a difference in relapses (any significant departure from an equal distribution) between seasonal groups (spring-summer/autumn-winter). A p-value less than 0.05 was considered statistically significant for all tests performed.

## Results

**Figure 1:**
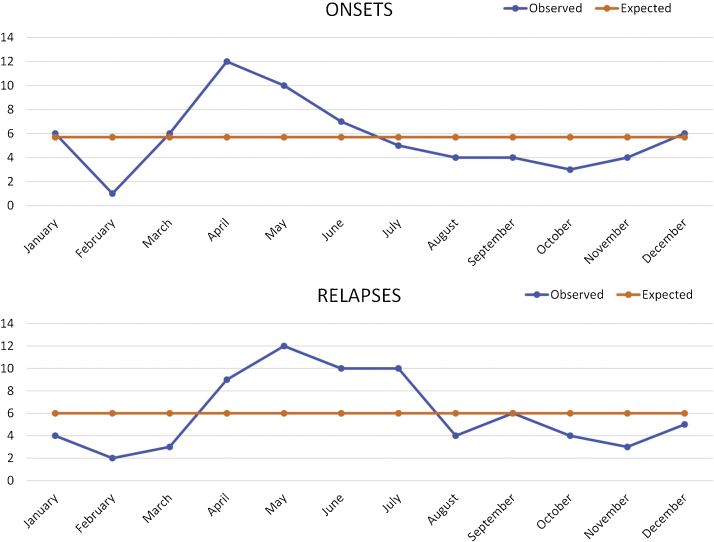
A. Monthly distribution of the onsets (χ2 = 20.67, p = 0.037). B. Monthly distribution of the subgroup of the relapses (without identified causes; χ2 = 13.96, p = 0.235).

A total of 68 PF patients (35 males, 33 females) were enrolled in the study. The mean age at diagnosis was 45.7 ± 14.5 years (median = 44; range, 20–75 years) with no significant difference between males (47.8 ± 14.3 years) and females (43.5 ± 14.5 years). The mean duration of symptoms prior to diagnosis was 1.7 ± 2.7 months (median = 1; range, 0.3–18 months). The distribution of the lesions was as follows: facial lesions in 27 patients, trunk lesions in 57 patients, limb lesions in 46 patients, and scalp lesions in 33 patients. While 4 of thepatients had localized lesions, others had lesions on multiple body parts. Eighteen patients (26.5%) were smokers and 22 patients (32.4%) had at least one comorbidity (diabetes mellitus in 9 patients, hypertension in 8 patients, hypothyroidism in 3 patients, and depression in 2 patients). When the professions of the patients were grouped, workers (n = 10), teachers (n = 7), and housewives (n = 24) were leading. The general characteristics of patients are summarized in Table 1. The number of patients diagnosed with PV within the study years was 408 (unpublished data) and the PV to PF ratio was calculated as 6. The number of onsets of the disease (according to the initial lesions) was the highest in April (n = 12) and lowest in February (n = 1) (Fig. 1a). The total number of onsets was found higher in spring-summer (n = 45) compared to autumn-winter (n = 23) (χ2 = 7.118, p = 0.008) (Table 2).

**Table 1:** General characteristics of pemphigus foliaceus patients.

**Patients, n**	68
**Male/Female, n (%)**	35 (51.5%) / 33 (48.5%)
**Mean age at the diagnosis (years)**	45.70 ± 14.47 (median = 44.5; range, 20–75)
**Mean time delay prior to diagnosis (months)**	1.7 ± 2.7 (median = 1; range, 0.3–18)
**Distribution of the lesions (localized / generalized)**, n (%)	4 (5.9%) / 64 (94.1%)
**Smokers, n (%)**	18 (26.5%)
**Patients with comorbidities, n (%)**	22 (32.4%)
**Mean follow up period (years)**	5.1 ± 3.1 (median = 5; range, 1–15)
**Patients with relapses**^a^**, n (%)**	42 (73.7%)
**Patients with multiple relapses**^a^**, n (%)**	29 (50.9%)
**Patients without relapses**^a^**, n (%)**	15 (26.3%)
**Patients with therapy-associated adverse events**^a^**, n (%)**	24 (42.1%)

a Ratio to the number of the patients with at least one year of follow-up (n = 57).

**Table 2:** Seasonal distribution of the onsets of pemphigus foliaceus and relapses.

Onsets of pemphigus foliaceus (n = 68)			
	Observed	Expected (68/2)	p-value
Spring–Summer	45	34	**p** = **0.008**
Autumn–Winter	23	34	

All relapses (n = 117)			
	Observed	Expected (117/2)	p–value
Spring–Summer	66	58.5	p = 0.166
Autumn–Winter	51	58.5	

Subgroup of relapses (without identified causes) (n = 72)			
	Observed	Expected (72/2)	p–value
Spring–Summer	48	36	p = 0.005
Autumn–Winter	24	36	

The mean follow-up period was calculated as 5.1 ± 3.1 years (median = 5; range, 1–15 years) in 57-patients with at least a one-year follow-up. Among these patients, systemic corticosteroids were used as the initial therapy agent and at least one adjuvant therapy agent (azathioprine, mycophenolic acid, dapsone, rituximab, and intravenous immunoglobulin) was started on 35 of them during the follow-up. Twenty-four patients experienced at least one adverse effect including diabetes mellitus (n = 10), hypertension (n = 7), osteoporosis (n = 9), cataracts (n = 7), acute kidney failure (n = 1), pneumonia (n = 1), abscess (n = 2), anemia (n = 1), pancytopenia (n = 1), and methemoglobinemia (n = 1) during their treatments.

Forty-two patients (24 male, 18 female) relapsed at least once during their follow-up and 29 of them experienced multiple relapses (twice in 11-patients; three times or more in 18-patients). A total of 117 relapses occurred in the present study’s series. The mean systemic corticosteroid dose used at the time of the relapses was calculated as 8.65 ± 10.55 mg (range, 4–30 mg). The most common daily dose at the time of the relapses was 4 mg (n = 23). During 21 of the relapses, the patients were not receiving any systemic corticosteroids. Only 5 patients relapsed while receiving an adjuvant therapy agent. There were no significant differences noted in terms of the sex of patients, smoking habit, distribution of the lesions, and existence of comorbidities between relapsed and non-relapsed patients (Table 3). Although mean age at the diagnosis and time delay prior to diagnosis was higher in the relapsed group (mean age, 46.1 ± 14.4 vs. 44.1 ± 14.6 years; time delay prior to diagnosis, 2 ± 3.5 vs. 1.4 ± 1.1 months), the differences were not statistically significant (Table 3). The rate of adjuvant therapy agent use was higher in relapsed patients compared to the non-relapsed patients with a statistical significance (OR = 4.462; 95% CI 1.269–15.684; p = 0.020) (Table 3). The number of relapses was the highest in May (n = 17) and lowest in February (n = 6). The total number of relapses was higher in spring-summer (n = 66) compared to autumn-winter (n = 51) (not significant; Table 2). The trigger factors of the 45 relapses could be identified as follows: respiratory tract infections (n = 18), misuse of treatment drugs (n = 11), stress (surgery, accidental injury, or psychological trauma**)** (n = 9), fasting (n = 3), and other infections (n = 4). The total observed number of relapses without identified causes was 72, and the highest in May (n = 12) and lowest in February (n = 2) (Fig. 1b). The total number of these relapses was higher in spring-summer (n = 48) compared to autumn-winter (n = 24) and it reached a statistical significance (χ2 = 8, p = 0.005) (Table 2).

**Table 3:** Binomial logistic regression analysis of variables between relapsed and non-relapsed patients.

Variable	Relapsed patients (n = 42)	Non-relapsed patients (n = 15)	p-value	OR	95% CI
Age, y (continuous)	46.1 ± 14.4	44.1 ± 14.6	0.641	0.990	0.949–1.033
Sex (male/female)	24/18	5/10	0.120	2.667	0.775–9.172
Time delay until diagnosis, m (continuous)	2.0 ± 3.5	1.4 ± 1.1	0.483	0.905	0.685–1.196
Smoking (yes/no)	13/29	2/13	0.197	2.914	0.573–14.814
Distribution of the lesions (generalized/localized)	41/1	13/2	0.146	6.308	0.528–75.337
Comorbidities (yes/no)	11/31	7/8	0.149	0.406	0.119–1.381
Use of adjuvant therapy agents (yes/no)	29/13	5/10	0.020	4.462	1.269–15.684

y, years; m, months; OR, Odds Ratio; CI, Confidence Interval.

## Discussion

In the current study, presented series of non-endemic PF patients is the largest in Turkey and one of the largest in the world (Table 4). The demographic features including, mean age at the diagnosis (45.7 ± 14.5 years), the ratio of male to female genders (1.1), and PV to PF subtypes (6) were generally consistent with current literature (Table 4).^19^, ^20^, ^22^, ^23^, ^24^, ^25^, ^26^, ^27^, ^28^, ^29^, ^30^, ^31^, ^32^, ^33^, ^34^, ^35^, ^36^ It was discovered that the onset of PF was more common in spring-summer comparing to autumn-winter in the present study’s series with a statistical significance; however, data of any detected trigger factors of the initial onsets were not available in the authors’ records. While winter was reported with the highest number of onsets in a group of 223 pemphigus patients (including 20 PF patients) in a study, spring-summer was reported to produce the highest number in a group of 74 pemphigus patients (including 15 PF patients) in another.^19^, ^20^ In a third study, the onset was more common in spring-summer in 222 PV patients, but the difference was not statistically significant.^21^

**Table 4:** Demographical pemphigus studies from non-endemic countries.

Author/Year	Country	Study period (years)	PV (n)	PF (n)	PV to PF ratio	Male to female ratio^a^	Mean age^a^
Current study, 2020	Turkey	29	408	68	6	1.1	46
Micali,^23^ 1998	Italy	15	63	14^b^	4.5	NA	54
Tsankov,^19^ 2000	Bulgaria	16	57	13	4.4	NA	NA
Tallab,^24^ 2001	Saudi Arabia	10	18	1^b^	18	NA	NA
Nanda,^25^ 2004	Kuwait	12	48	11	4.4	0.4	37
Chams-Davatchi,^26^ 2005	Iran	20	1111	89	12.5	NA	NA
Golusin,^27^ 2005	Serbia	13	37	6	6.2	NA	NA
Uzun,^28^ 2006	Turkey	7	123	13	9.5	1.2	52
Salmanpour,^20^ 2006	Iran	10	194	20	9.7	1.2	NA
V’lckova-Laskoska, ^29^ 2007	Macedonia	15	103	21	4.9	NA	NA
Kumar,^30^ 2008	India	1	10	2	5	1	46
Marazza,^31^ 2009	Switzerland	2	4	3	1.3	NA	NA
Baican,^32^ 2010	Romania	7	55	9	6.1	NA	NA
Kulthanan,^33^ 2010	Thailand	18	98	19	5.2	0.5	57
Bozdağ,^34^ 2012	Turkey	12	81	6	13.5	NA	NA
Kridin,^35^ 2016	Israel	6	159	20	8	1.9	NA
Milinkovic,^36^ 2016	Serbia	20	384	86	4.5	NA	NA

PV, Pemphigus Vulgaris; PF, Pemphigus Foliaceus; NA, Not Applicable.

a PF.

b PF/Pemphigus erythematosus.

Although PF is known as a milder disease compared to PV, relapsing is not a risk to be ignored. Forty-two patients (74%) of the present study’s series relapsed at least once and 29 patients (51%) experienced it more than once. Each relapse required an increase in the dose of the systemic corticosteroids and/or a new intervention with an adjuvant agent. There were no significant differences noted in terms of the sex of patients, age at the diagnosis, smoking habit, time delay prior to diagnosis, distribution of the lesions, and presence of comorbidities between relapsed and non-relapsed patients. Although the rate of the adjuvant therapy agent use was higher in the relapsed group with a statistical significance (OR = 4.462; 95% CI 1.269–15.684; p = 0.020), no further comment about the relation of adjuvant therapies and relapses can be made due to the lack of disease severity data in the present study.

When the seasonal distribution of all relapses (n = 117) was analyzed, the number of relapses reached its peak in spring-summer but without a statistical significance. UV radiation from sunlight exposure was shown to induce or aggravate several autoimmune blistering diseases such as PV, PF, linear IgA dermatosis, and bullous pemphigoid in the previous studies.^10^, ^16^ Controlled UVB exposure was reported to lead acantholysis on the intact skin of PV and PF patients; whereas, this was negative in the skin of the control patients.^14^ These data put forward the hypothesis that a UV mediated-seasonal variation may exist in the course of bullous diseases.^21^ However, UV is not an easy-to-detect factor in relapses and was not a recorded cause in the authors’ records. There were common trigger factors such as infections, misuse of the drugs, and fasting among the studied patients. Infections, particularly respiratory tract infections, are easy-to-detect trigger factors by anamnesis, physical examination, and laboratory testing and are more common in autumn-winter; however, they were detected in less than half of the relapses in the present study. In order to determine the role of UV radiation as a hidden trigger, 72 relapses for which a trigger factor could not be detected were analyzed and they showed a significant seasonal variation with being the most common in spring-summer. The only other study found in the literature investigating the seasonal variation in pemphigus relapses was performed on a group of PV patients (Table 5).^14^ Unlike the presented study, the first relapse and the second relapse experienced by patients were studied in different groups. Although summer ranked first in initial and subsequent relapse, the difference was not statistically significant. Based on the authors’ experience of daily practice, the influence of UV may be stronger in PF patients than in PV patients; yet, no comparative study has been found in the literature for the subtypes of pemphigus. To support this view, studies on large series of PF and PV patients are needed.

**Table 5:** Studies on seasonal variations of pemphigus.

Reference	Patients	Peak season of the onsets (statistical significance)	Peak season of the relapses (statistical significance)
Current study	68 PF	Spring–Summer (Significant)	Spring–Summer (Not significant in the total group; Significant in the subgroup)
Tsankov et al.^19^	57 PV	Spring–Summer (NA)	NA
	15 PF		
	2 PH		
Salmonpour et al.^20^	194 PV	Winter (NA)	NA
	20 PF		
	9 PH		
Robati et al.^21^	222 PV	Summer (Not significant)	Spring (Not significant)

PF, Pemphigus Foliaceus; PV, Pemphigus Vulgaris; PH, Pemphigus Herpetiformis; NA, Not Applicable.

The main limitation of the study was its retrospective design.

## Conclusion

Non-endemic PF is a very rare disease with a variable clinical course that may present with relapses and management of the relapses is not always easy due to hidden triggers. The study’s records showed that trigger factors could not be identified in more than half of the relapses in the study’s series. The subgroup of the relapses that were with hidden triggers as well as the initial onsets of the disease showed a significant seasonal variation with a peak in spring-summer; however, the seasonal variable did not justify the total group of relapses in our series. Although the shown seasonal variation may be caused due to a combination of factors, the authors believe that UV radiation should be considered an important trigger factor for the peak in spring-summer seasons, particularly in Turkey. Although the mortality rate of pemphigus has been low due to treatment with systemic corticosteroids and other therapeutic agents in the last decades, understanding the preventable factors of relapses is critical due to the remarkable number of therapy-associated morbidities.^37^ It is advisable for clinicians to emphasize the importance of protection from UV to the patients in geographical regions like Turkey where spring-summer seasons are characterized by intense UV radiation. Furthermore, this factor should be taken into consideration in treatment plans during the transition to spring-summer.

## Ethical statement

This study was performed in line with the principles of the Declaration of Helsinki. Approval was granted by the Ethics Committee of İstanbul University (20.10.2016/363903).

## Financial support

None declared.

## Author’s contribution

Sıla Kılıç Sayar: Conceptualization; data curation; formal analysis; investigation; methodology; software; supervision; visualization; writing e draft; writing e editing.

Rıfkiye Küçükoğlu: conceptualization; data curation; formal analysis; investigation; methodology; software; supervision; visualization; writing e draft; writing-review e editing.

## Conflicts of interest

None declared.
